# The mechanism of thia-Michael addition catalyzed by LanC enzymes

**DOI:** 10.1073/pnas.2217523120

**Published:** 2023-01-12

**Authors:** Chayanid Ongpipattanakul, Shi Liu, Youran Luo, Satish K. Nair, Wilfred A. van der Donk

**Affiliations:** ^a^Department of Biochemistry, University of Illinois at Urbana-Champaign, Urbana, IL 61801; ^b^Department of Chemistry, University of Illinois at Urbana-Champaign, Urbana, IL 61801; ^c^Carl R. Woese Institute for Genomic Biology, University of Illinois at Urbana-Champaign, Urbana, IL 61801; ^d^HHMI, University of Illinois at Urbana-Champaign, Urbana, IL 61801; ^e^Center for Biophysics and Computational Biology, University of Illinois at Urbana-Champaign, Urbana, IL 61801

**Keywords:** lantibiotics, nisin, *C*-glutathionylation, Michael addition, dehydrobutyrine

## Abstract

In both eukarya and bacteria, the addition of Cys to dehydroalanine (Dha) and dehydrobutyrine (Dhb) occurs in various biological processes. In bacteria, intramolecular thia-Michael addition catalyzed by lanthipeptide cyclases (LanC) proteins or protein domains gives rise to a class of natural products called lanthipeptides. In eukarya, dehydroamino acids in signaling proteins are introduced by effector proteins produced by pathogens like *Salmonella* to dysregulate host defense mechanisms. A eukaryotic LanC-like (LanCL) enzyme catalyzes the addition of Cys in glutathione to Dha/Dhb to protect the cellular proteome from unwanted chemical and biological activity. To date, the mechanism of the enzyme-catalyzed thia-Michael addition has remained elusive. We report here the crystal structures of the human LanCL1 enzyme complexed with different ligands, including the product of thia-Michael addition of glutathione to a Dhb-containing peptide that represents the activation loop of Erk. The structures show that a zinc ion activates the Cys thiolate for nucleophilic attack and that a conserved His is poised to protonate the enolate intermediate to achieve a net *anti-*addition. A second His hydrogen bonds to the carbonyl oxygen of the former Dhb and may stabilize the negative charge that builds up on this oxygen atom in the enolate intermediate. Surprisingly, the latter His is not conserved in orthologous enzymes that catalyze thia-Michael addition to Dha/Dhb. Eukaryotic LanCLs contain a His, whereas bacterial stand-alone LanCs have a Tyr residue, and LanM enzymes that have LanC-like domains have a Lys, Asn, or His residue. Mutational and binding studies support the importance of these residues for catalysis.

## Significance

The addition of the thiol side chain of Cys residues to dehydroalanine and dehydrobutyrine is a process that occurs in all domains of life and is catalyzed by LanC enzymes. In bacteria, this process is intramolecular resulting in macrocyclic structures called lanthipeptides that have antimicrobial, antiviral, morphogenetic, and virulence activities. In eukarya, the process is intermolecular and involves the addition of the thiol of glutathione to remove electrophilic structures from the proteome, thus preventing dysregulation of cellular processes. This work reports the cocrystal structures of the human LanCL1 enzyme and the glutathione adduct to a Dhb-containing peptide. The structure provides key insights into the mechanism of catalysis and may allow design of inhibitors as well as catalysts for synthetic biology.

**Fig. 1.**
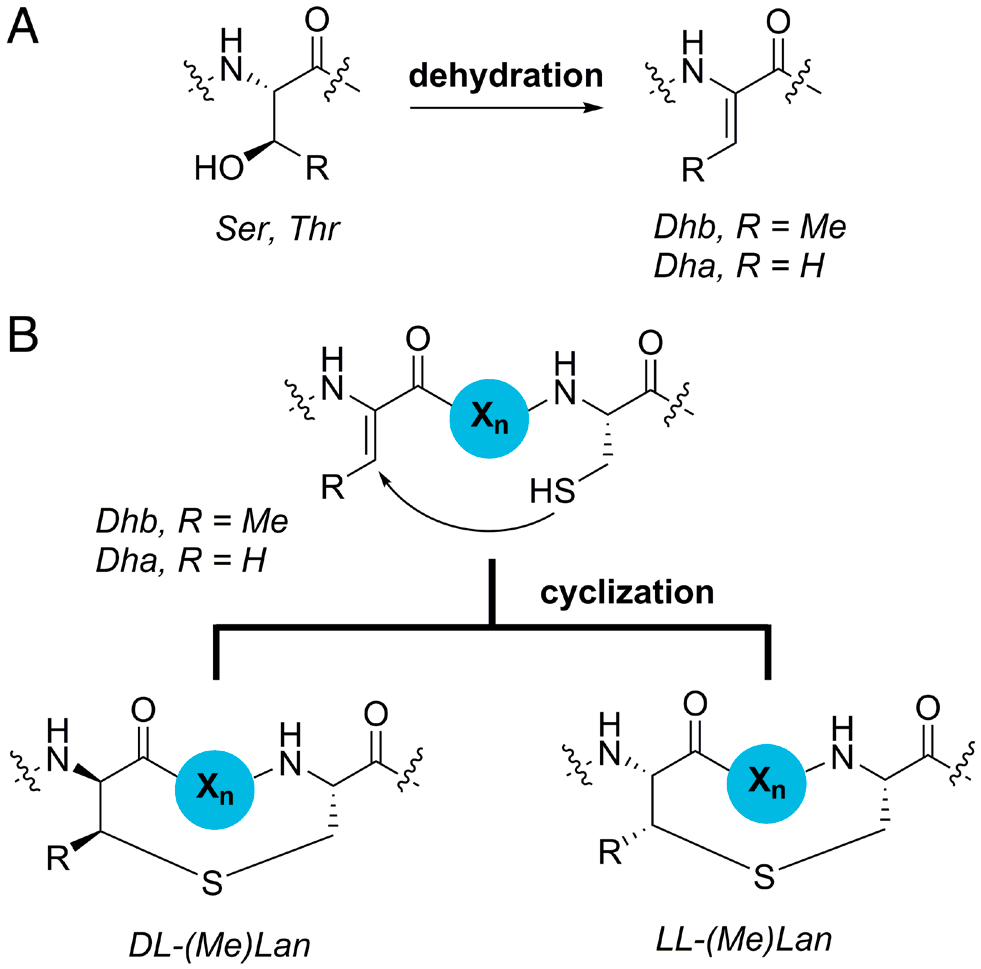
(*A*) Generation of Dha by serine dehydration. (*B*) Formation of macrocycles by intramolecular addition of cysteine to Dha/Dhb residues. X_n_ = peptide chain.

Dehydroamino acids are reactive structures involved in enzymatic catalysis (1, 2), natural product biosynthesis (3–5), aging (6), and infection (7). The most common dehydroamino acids are dehydroalanine (Dha, Fig. 1*A*) and dehydrobutyrine (Dhb), formed from the dehydration of Ser and Thr residues, respectively. In bacteria, Dha and Dhb are found in numerous natural products including members of the ribosomally synthesized and post-translationally modified peptides (5). For example, enzyme-catalyzed dehydration reactions of Ser/Thr generate Dha/Dhb in the final structures and/or biosynthetic intermediates of thiopeptides and lanthipeptides (8). In the case of lanthipeptides, the intramolecular addition of Cys residues to the Dha/Dhb residues results in the formation of macrocycles containing lanthionine (from Dha) or methyllanthionine (from Dhb) (Fig. 1*B*) (3). These conformationally restricted polycyclic peptides possess a variety of bioactivities including antimicrobial [lantibiotics; e.g., the commercial food preservative nisin (9)], antiviral (duramycin and its derivatives (10, 11)), and virulence [the enterococcal cytolysin (12)]. Whereas the dehydration step in lanthipeptide biosynthesis is relatively well understood at the structural and biochemical level (13–15), the mechanistic details of the cyclization step are much less clear.

Class I, II, and IV lanthipeptide cyclases or cyclase domains all have a similar α,α-toroidal fold (3). The prototype enzymes are class I lanthipeptide cyclases (LanCs) such as NisC involved in nisin cyclization (16). Class II and IV lanthipeptides are made by bifunctional enzymes that possess dehydratase domains joined to cyclase domains. The cyclase domains of these two bifunctional enzyme classes are structurally related to the stand-alone LanCs. These cyclases each contain a zinc ion that is believed to activate the substrate thiols for attack onto the dehydroamino acids, but direct experimental evidence for this role is not available. Similarly, the mechanism of recognition of the dehydroamino acids is not known.

In higher organisms, Dha and Dhb are generated either during aging through chemical damage to proteins at sites of Ser/Thr phosphorylation (6), or by enzyme-catalyzed elimination of the phosphate of pSer or pThr. The latter strategy is used by pathogens like *Salmonella* and *Shigella*, which translocate specific effector proteins into host cells to target activated kinases in the mitogen-activated signal transduction pathway (17–19). Effector-catalyzed phosphate elimination of pSer/pThr in the activation loop of these kinases is an irreversible process that derails control of their activity by kinase and phosphatase regulation. Mammals have three LanC-like proteins (LanCL1-3) that were recently shown to catalyze intermolecular addition of the thiol of glutathione (GSH) to Dha and Dhb in a variety of proteins (20). The addition of GSH (*C*-glutathionylation) removes electrophilic Dha/Dhb residues from the proteome.

A better understanding of the chemical mechanism of the LanC-catalyzed addition of thiols to Dha/Dhb would aid in engineering the biosynthesis of lanthipeptides and tailor-made non-natural peptides (5), in potential enzymatic modification of Dha/Dhb containing proteins (21, 22), and in designing potential inhibitors of the biosynthesis of natural products that are detrimental to human health like cytolysin (12). We present herein crystal structures of human LanCl1 bound to methyl GSH (1.58 Å resolution) or to the product of GSH addition to a Dhb-containing peptide that mimics the activation loop of Erk (1.91 Å resolution). The structures guided mutational analysis of residues that may function in catalysis. Our studies reveal previously unknown aspects regarding substrate recognition and activation as well as unexpected differences in the enzymes that catalyze thia-Michael additions.

## Results

### Cocrystal Structure of LanCL1 Bound to a GSH-Addition Product.

Previous structures of NisC (16), LanCL1 (23), LanCL2 (20), and the LanC-domain containing CylM (14) all show a characteristic α,α-toroid fold with a zinc ion bound at the top of the barrel by two His residues and a Cys residue. The only cocrystal structure is that of LanCL1 bound to GSH (23), which shows that the Cys sulfur of GSH completes the four-coordinate tetrahedral geometry around the zinc ion. Thiolate coordination to the zinc ion supported a previous proposal that the role of the zinc ion is to activate the nucleophile (Cys thiolate) and not the carbonyl group of the electrophile (Dha/Dhb) (24). The quality of this published 2.8 Å resolution co-crystal structure is of concern as a) the carbonyl carbon atoms of GSH are refined with tetrahedral geometry and b) the amide bonds of the ligand are non-planar. Moreover, the question as to how the LanC enzymes recognize and activate the Dha/Dhb residues has yet to be addressed.

In this study, we first determined the co-crystal structures of LanCL1 with GSH (to 1.51 Å), and with *S-*methyl GSH (hereafter MeGSH, to 1.58 Å). The binding poses of both GSH and MeGSH differ from that observed in the prior low-resolution structure of LanCL1 and density for the amide bonds are consistent with their expected planar configuration (*SI Appendix*, Fig. S1). We next set out to determine the structure of LanCL1 in complex with GSH and a (*Z*)-Dhb-containing 13-mer peptide (DHTGFL-Dhb-EYVATR) corresponding to the sequence of the activation loop of Erk (1.91 Å resolution). A phosphoThr (pThr) in this loop is the target of the pThr lyases SpvC and OspF that catalyze phosphate elimination to generate (*Z*)-Dhb (17–19). We recently showed that LanCL1 and LanCL2 add GSH to this peptide (20). We used solid-phase peptide synthesis to first prepare the Erk peptide with a pThr and then used recombinant SpvC to convert the peptide to the Dhb-containing product.

**Fig. 2.**
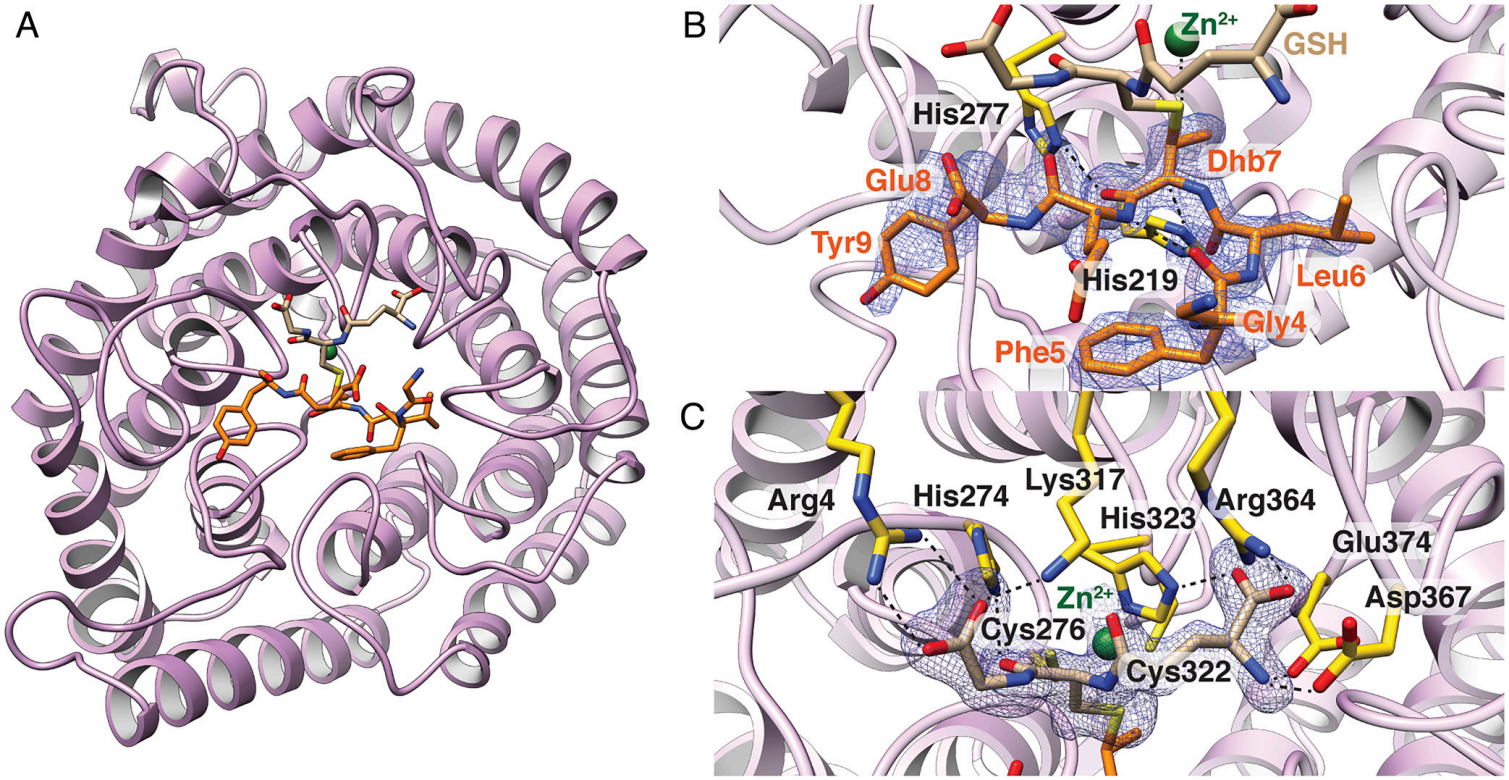
(*A*) Crystal structure of LanCL1 bound to the addition product of GSH and Dhb-Erk peptide. The difference Fourier map (F_o_−F_c_), calculated with the coordinates of ligand omitted for one cycle of refinement, is shown in blue and contoured at 3σ above background. (*B*) Close-up of the interactions between LanCL1 residues (gold carbon atoms) and the Dhb-Erk peptide (orange carbon atoms). The catalytic Zn^2+^ is shown as a green sphere. The numbering of Dhb-Erk corresponds to the residue number within the synthetic 13-mer peptide. Although the amino acid at position 7 is labeled as Dhb, this residue has reacted with GSH to become part of a methyllanthionine. (*C*) Close-up of the interactions between LanCL1 residues (yellow carbon atoms) and GSH (tan carbon atoms). LanCL1 residues Cys276, His323, and Cys322, along with the thiolate of GSH coordinate the Zn^2+^.

To obtain crystal structures of LanCL1 bound to GSH and its peptide substrate, the cocrystals of LanCL1 with GSH were soaked with Dhb-Erk for various time points. Prolonged incubation revealed that Cys264, located distal to the active site, appeared to form an adduct with GSH (*SI Appendix,* Fig. S2). We therefore determined the structure of LanCL1-C264A in complex with GSH (1.79 Å resolution), showing that the structure is near-identical to that of the wild-type enzyme (*SI Appendix,* Fig. S3). The mutation of this Cys residue to Ala only moderately affected the activity of the enzyme (Table 1). Hence, to rule out off-target GSH binding effects, all mutants discussed in kinetic and binding studies are in the background of the Cys264Ala mutation. The optimization of peptide soaking duration resulted in the structure of native LanCL1 with electron density at the active site consistent with the presence of GSH in a covalent complex with Dhb-Erk (Fig. 2*A*). Density was only observed for the GFL-Dhb-EY segment of the substrate peptide, which is clearly connected to the GSH molecule (distance between the former GSH sulfur atom and former Dhb β-carbon is 1.91 Å).

**Table 1.** Kinetic parameters for the addition of GSH to (*Z* )-Dhb-Erk peptide

Protein	*k*_cat_ (min^−1^)	*K*_m,peptide_ (μM)	*k*_cat_/*K*_m,peptide_ (M^−1^ min^−1^)
LanCL1 Wild type	7.5 ± 0.2	96 ± 9.0	(7.8 ± 0.8) × 10^4^
LanCL1 C264A	2.7 ± 0.1	85 ± 7.0	(3.2 ± 0.3) × 10^4^
LanCL1 C264A/H277N	17.2 ± 2.2	417 ± 76	(4.1 ± 0.9) × 10^4^
LanCL1 C264A/H277Y	7.84 ± 0.18	69 ± 5.0	(1.1 ± 0.1) × 10^5^
LanCL1 C264A/H277A	1.72 ± 0.10	240 ± 30	(7.2 ± 1.0) × 10^3^
LanCL1 C264A/H277D	–^*^	–^^*^^	(3.7 ± 0.1) × 10^2^
LanCL1 C264A/H277K	–^^*^^	–^^*^^	(1.6 ± 0.3) × 10^2^

^*^Substrate saturation could not be achieved for these mutants. The Michaelis–Menten curves for all enzymes are shown in *SI Appendix,* Fig. S6.

The inspection of the non-hydrogen atoms attached to the Cα and Cβ of the former Dhb residue in the peptide indicated that both carbons have tetrahedral geometry suggesting that the crystals contain the product of the thia-Michael addition of GSH to Dhb-Erk peptide. Modeling of the electron density is most consistent with a product complex (Fig. 2*B* and *SI Appendix,* Fig. S4), and in agreement with the experimentally determined stereochemistry of the LanCL1 catalyzed addition reaction, which generates 2*S*,3*S*,6*R* methyllanthionine (DL-MeLan, Fig. 1*B*) (20). This stereochemistry indicates an *anti-*addition to the (*Z*)-Dhb. We have previously suggested that a conserved His in the active site may serve as the active site acid that protonates the enolate intermediate to set the 2*S* stereochemistry (16, 25). Indeed, Nε2 of this His residue (His219 in LanCL1) is 3.7 Å from the α-carbon of the former Dhb, consistent with the proposed role in catalysis (Fig. 2*B*). The distance between the thioether sulfur atom and the zinc ion is 2.7 Å (Fig. 2*B*) suggesting that the sulfur was indeed bound to the zinc ion as the thia-Michael addition progressed. The most unexpected observation revealed by the co-crystal structure is a hydrogen bond between the carbonyl carbon of the former Dhb and Nε2 of His277 (distance of 2.8 Å) (Fig. 2*B*). Attempts to prevent the reaction in the crystal by using MeGSH during co-crystallization did not result in a ternary complex, likely because the methyl group on MeGSH prevents peptide binding (*SI Appendix,* Fig. S5).

Other than the hydrogen bond between His277 and the carbonyl oxygen, no other obvious contacts are observed between the peptide product and LanCL1, consistent with the high substrate tolerance of LanCL1 shown in a previous study (20). As expected, the GSH part of the methyllanthionine crosslinked product is recognized by several highly specific interactions. LanCL1 residues Arg4 and Lys317 form hydrogen bonds with the Gly-derived portion of GSH, His274 engages the Cys-derived amide carbonyl, and His323, Arg364, Asp367, and Glu374 make hydrogen bonds with the Glu-derived terminus of GSH (Fig. 2*C*). The same interactions are present in the structure of LanCL1 bound to MeGSH (*SI Appendix,* Fig. S5).

### His277 Is Not Conserved in LanC-Type Enzymes.

**Fig. 3.**
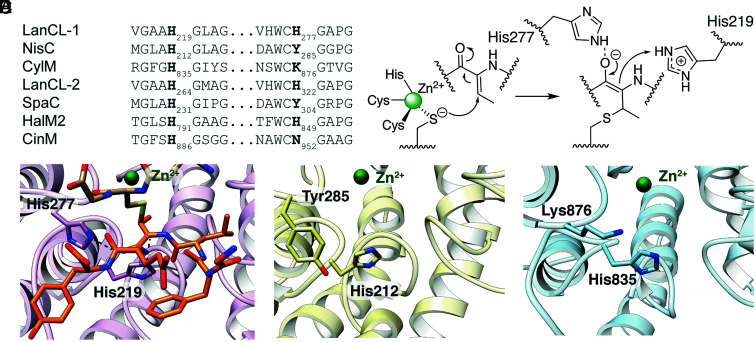
Residues stabilizing the enolate intermediate in thia-Michael addition of LanC, LanCL, and LanM enzymes. (*A*) Multiple sequence alignment of the relevant regions of LanC, LanCL, and LanM enzymes that illustrate the conserved nature of the active site acid (His219 in LanCL1) that protonates the enolate intermediate, and the substitution of His277 with other amino acids in various related enzymes. (*B*) Proposed mechanism of the thia-Michael addition in which a zinc ion lowers the p*K*_a_ of a coordinated Cys thiol and activates the resulting thiolate for nucleophilic addition to Dhb and a protonated His277 stabilizes the enolate oxygen in the intermediate. The imidazolium side chain of His219 then protonates the enolate at the α-carbon. His277 is shown in neutral form and His219 in protonated form, but the protonation state of neither is known.(*C*) Close-up of the product of the thia-Michael addition of GSH to Dhb-Erk peptide bound to LanCL1 illustrating the position of His277 and His219 with respect to the product peptide. (*D*) Positions of Tyr285 and His212 in the structure of NisC (PDB ID 2G0D) that are analogous to His277 and His219 in LanCL1. (*E*) Positions of Lys876 and His835 in the cyclase domain of the structure of CylM (PDB ID 5DZT) that are analogous to His277 and His219 in LanCL1.

Multiple sequence alignments of LanC and LanC-like proteins show that His277 is not conserved (Fig. 3*A* and *SI Appendix,* Table S1), which is why this residue has been overlooked in previous studies. In eukaryotic LanCL proteins, the corresponding residue is always a His, but in bacterial LanC enzymes, this residue is predominantly a Tyr. In the LanC-like domains of class II lanthipeptide synthetases (LanMs), this residue is either a His [e.g., in ProcM (26) or HalM2 (27)], a Lys [e.g., CylM involved in cytolysin biosynthesis (14)], or an Asn (e.g., CinM/DurM (28, 29) involved in biosynthesis of duramycin-like peptides). The overlay of this region of interest of the structures of NisC (bacterial LanC), the cyclase domain of CylM, and the current structure of LanCL1 shows that Tyr285 of NisC and Lys876 of CylM indeed occupy the same space as His277 of LanCL1 (Fig. 3 *C*–*E*), suggesting these residues could serve a similar role of activating the Dhb carbonyl oxygen. Thus, although the residues involved vary considerably, a general strategy of a side chain functional group that can stabilize the enolate oxygen emerges.

### Site-Directed Mutagenesis Studies on His277.

Based on the observed structure, we conducted mutagenesis studies on His277 of LanCL1, converting this residue to Ala, as well as to the amino acids that are seen in the alignment in Fig. 3*A*. As a test of the interaction of this residue with the enolate oxygen during catalysis, the His277Asp variant was also prepared. All variants were made in the context of the Cys264Ala background such that potential disulfide formation with GSH would not affect the kinetic profiles. All mutant enzymes were expressed in *Escherichia coli* as N-terminally His_6_-tagged proteins and were purified by nickel affinity chromatography. The initial rates of GSH addition to Dhb-Erk were determined by LC-MS as a function of peptide concentration at saturating concentrations of GSH (*SI Appendix,* Fig. S6) (20). We used Dhb-Erk rather than Dha-Erk in these experiments to avoid the background non-enzymatic thia-Michael addition by GSH that has been observed for Dha-Erk (20); non-enzymatic addition to Dhb is very slow (20). Fitting of the observed rates to the Michaelis–Menten equation provided *k*_cat_ and *K*_m,peptide_ values shown in Table 1.

The mutation of His277 to Ala surprisingly led to only a modest decrease in *k*_cat_ suggesting that the ability to stabilize the enolate intermediate is not critical for catalysis. The mutation led to an increase in *K*_m,peptide_ suggesting His277 participates in peptide substrate recognition (Table 1 and *SI Appendix,* Fig. S6). Thus, the catalytic efficiency (*k*_cat_/*K*_m_) of the variant is reduced tenfold. The mutation of His277 to residues found in other LanC-type enzymes had varying effects. The replacement with Tyr, as found in bacterial LanC proteins, did not significantly change *k*_cat_/*K*_m,peptide_, whereas replacement with Lys, as found in a subset of class II LanM cyclase domains, greatly affected catalysis such that saturation of the substrate could no longer be achieved (*SI Appendix*, Fig. S6). The decrease in *k*_cat_/*K*_m,peptide_ is about 200-fold compared to the parent Cys264Ala mutant, suggesting that the Lys side chain is not able to effectively accommodate the substrate. A similarly large effect was observed upon substitution of His277 with a negatively charged residue (Asp), likely because of repulsion between the side chain carboxylate and the carbonyl/enolate oxygen of substrate/intermediate. Conversely, the replacement of His277 with Asn increased *k_cat_*, but also significantly increased *K*_m,peptide_ (Table 1). Collectively, these data suggest that His277 is moderately important for catalysis and appears to contribute to substrate recognition.

### Binding Studies of Dha-Erk Peptide and GSH with LanCL1 and Its Variants.

The co-crystal structure as well as previously determined *K*_m_ value for GSH (~30 μM) (20) suggests that LanCL1 first binds GSH and is saturated with GSH in the cellular environment. To corroborate this hypothesis, we determined the affinity of LanCL1 for GSH by isothermal titration calorimetry (ITC), providing a *K*_D,GSH_ of 9.6 ± 0.6 μM (*SI Appendix,* Fig. S7). We also carried out binding analyses for LanCL2, as well as the bacterial NisC and HalM2 enzymes, where the latter two enzymes are not expected to, and indeed did not, bind GSH (*SI Appendix,* Fig. S7).

Next, we determined the binding affinity of LanCL1 and its variants for the Erk peptide containing a dehydroamino acid. N-terminally fluorescein-labeled Dha-Erk was prepared by solid-phase peptide synthesis using established methods to introduce the Dha from Cys (30). The affinity of LanCL1 and its variants for this fluorescently labeled peptide was determined by fluorescence polarization measurements. WT LanCL1 bound the peptide with a *K*_D_ of 19.3 ± 1.8 μM (Table 2 and *SI Appendix,* Fig. S8). The LanCL1-C264A variant that was used in the kinetic experiments displayed a similar, slightly increased *K*_D_. The substitution of His277 with Ala resulted in a ~sixfold increase in *K*_D_, whereas substitution with Tyr decreased the *K*_D_ of the Dha-Erk peptide (Table 2). The *K*_D_ of Dha-Erk peptide was strongly increased for the variants that also displayed greatly elevated *K*_m_ values (LanCL1-H277K and LanCL1-H277N) and binding could not be observed for LanCL1-H277D. These trends mirror the *K*_m_ values in Table 1. We note that, unlike the kinetic studies, all binding studies were performed in the absence of GSH to avoid catalytic turnover. We next evaluated the importance of the Dha structure for binding. Fluorescein-labeled Erk peptide in which the Dha was substituted by L-Ala bound fourfold weaker than Dha-Erk (*SI Appendix,* Table S4). This difference was even more pronounced in competition assays in which synthetic fluorescein-labeled Dha-Erk was displaced by Ac-Dha-Erk or Ac-Ala-Erk (*SI Appendix,* Table S5). These latter peptides that do not carry a large fluorescent group likely provide a better mimic of protein substrates.

**Table 2.** Binding affinity of LanCL1 and its variants for Flu-Dha-Erk determined by fluorescent polarization

Protein	*K*_d_ (μM)
LanCL1 Wild Type	19.3 ± 1.8
LanCL1 C264A	28.7 ± 3.9
LanCL1 C264A/H277N	315 ± 42
LanCL1 C264A/H277Y	11.0 ± 1.0
LanCL1 C264A/H277A	112 ± 20
LanCL1 C264A/H277D	–^^*^^
LanCL1 C264A/H277K	471 ± 160

^*^Binding was not observed. The binding curves for all enzymes are shown in *SI Appendix,* Fig. S8.

## Discussion

In this work, we report mechanistic details of thia-Michael-type addition of Cys residues to dehydroamino acids catalyzed by LanC-type enzymes. Based on commonality with other enzymes that activate thiol nucleophiles (31, 32), the zinc ion had been suggested to activate the thiol nucleophile, both by lowering its p*K*_a_ and by activating the resulting thiolate bound to the zinc ion. This role of the metal ion is supported by GSH binding with its thiolate coordinated to the zinc as observed in prior (23) and our current LanCL1 crystal structures. We additionally provide molecular details showing how the product peptide is bound to the enzyme with the thioether sulfur still situated near the zinc ion. Collectively, these observations strongly support that the zinc ion orients and activates the thiolate nucleophile.

Beyond the mechanism of nucleophile positioning, hypotheses regarding how LanC-type enzymes facilitate the conjugate addition have been based on indirect studies. The mutation of the His corresponding to His219 in LanCL1 had deleterious effects on catalysis in NisC (24), SpaC (33), LctM (34), ProcM (35), and LanCL1 and LanCL2 (20). In addition, the reverse (retro-Michael) reaction was abolished upon replacement of this residue (25). Thus, the amino acid was tentatively assigned the role of active site acid that protonates the enolate intermediate. The current structural data confirms this role as the Nε2 of His219 is the only side chain of the enzyme that is in a correct position to protonate the enolate during catalysis.

One important unresolved question has been how these enzymes recognize the electrophilic dehydroamino acids and how they stabilize the enolate intermediate given that the first step of a thia-Michael-type addition is endergonic (36). The current study illustrates why the use of sequence conservation did not resolve this question as a variety of amino acid side chains appear to achieve enolate stabilization in different enzymes. In LanCL1, His277 is hydrogen bonded to the carbonyl oxygen of the methyllanthionine product after conjugate addition of GSH to Dhb-Erk. Thus, this interaction likely stabilizes the high-energy intermediate enolate. His277 is conserved in eukaryotic LanCLs and is also present in a subset of LanC-type cyclase domains of class II LanM synthetases (*SI Appendix,* Table S1). This finding agrees with a previous bioinformatic study that showed that the eukaryotic LanCLs are phylogenetically closer to the cyclase domains of LanMs than to the bacterial class I LanCs (37). However, a substantial subset of LanM cyclase domains has His277 replaced by Lys or Asn (*SI Appendix,* Table S1). Only one of these proteins has been structurally characterized, CylM involved in the biosynthesis of the enterococcal virulence factor cytolysin (14). In CylM, Lys876 occupies an equivalent position in both sequence and structure to His277 suggesting that this residue may also stabilize the enolate. The mutation of His277 to Lys in LanCL1 resulted in a strong negative effect on catalysis and substrate binding affinity suggesting that within the LanCL1 fold, the longer side chain of Lys compared to His cannot be accommodated in a productive manner. For the majority of bacterial LanC enzymes, His277 is replaced by a Tyr residue such as in the structurally characterized NisC. This Tyr is again in the correct position to stabilize the enolate intermediate by hydrogen bonding of the phenolic hydrogen to the enolate oxygen (Fig. 3*D*). Indeed, LanCL1-H277Y displayed high catalytic activity and binding affinity toward the substrate.

The current structure containing product provides an explanation as to how LanCL1 and LanCL2 can add GSH to a wide variety of sequences, apparently having no selectivity for the residues that flank the dehydroamino acids in the substrates (20). In the cocrystal structure with the Dhb-Erk product bound, the amino acids flanking the methyllanthionine (Leu and Glu) both turn away from the active site and do not make any specific interactions with the enzyme, nor do the amino acids that are two residues N- and C-terminal from the methyllanthionine (Phe and Tyr). An intramolecular hydrogen bond between the carbonyl amide oxygen of Phe and the amide N-H of Glu (3.4 Å) of the product peptide stabilizes the observed binding pose and resembles a β-turn. Dehydroamino acids are known to conformationally induce turns (38) and the current data suggest that recognition of substrates may be through a combination of the Dha/Dhb interacting with His277 and a β-turn induced by the dehydroamino acid that positions the electrophile correctly for catalysis.

In this model, catalysis occurs by a combination of interactions that promote the reaction, including increasing the reactivity of the nucleophile by the zinc ion (39, 40), activation of the dehydroamino acid by His277, recognition of the β-turn that presents the dehydroamino acid at the tip of the turn, and positioning of both the nucleophile and electrophile. Removing one of these catalytic strategies by substitution of His277 with Ala may still leave the enzyme with considerable abilities to catalyze the thia-Michael addition explaining why the *k*_cat_ of LanCL1-C264A/H277A is not much affected. An alternative explanation is that addition to Cβ and protonation at the α-position is concerted such that no intermediate enolate is formed, but theoretical studies suggest that the barrier for such a process is much higher than for a stepwise reaction (36). The product structure suggests that GSH likely binds first to the enzyme as it is bound deeper into the active site. GSH binding to LanCL1 in the absence of peptides is also supported by the ITC experiments.

**Fig. 4.**
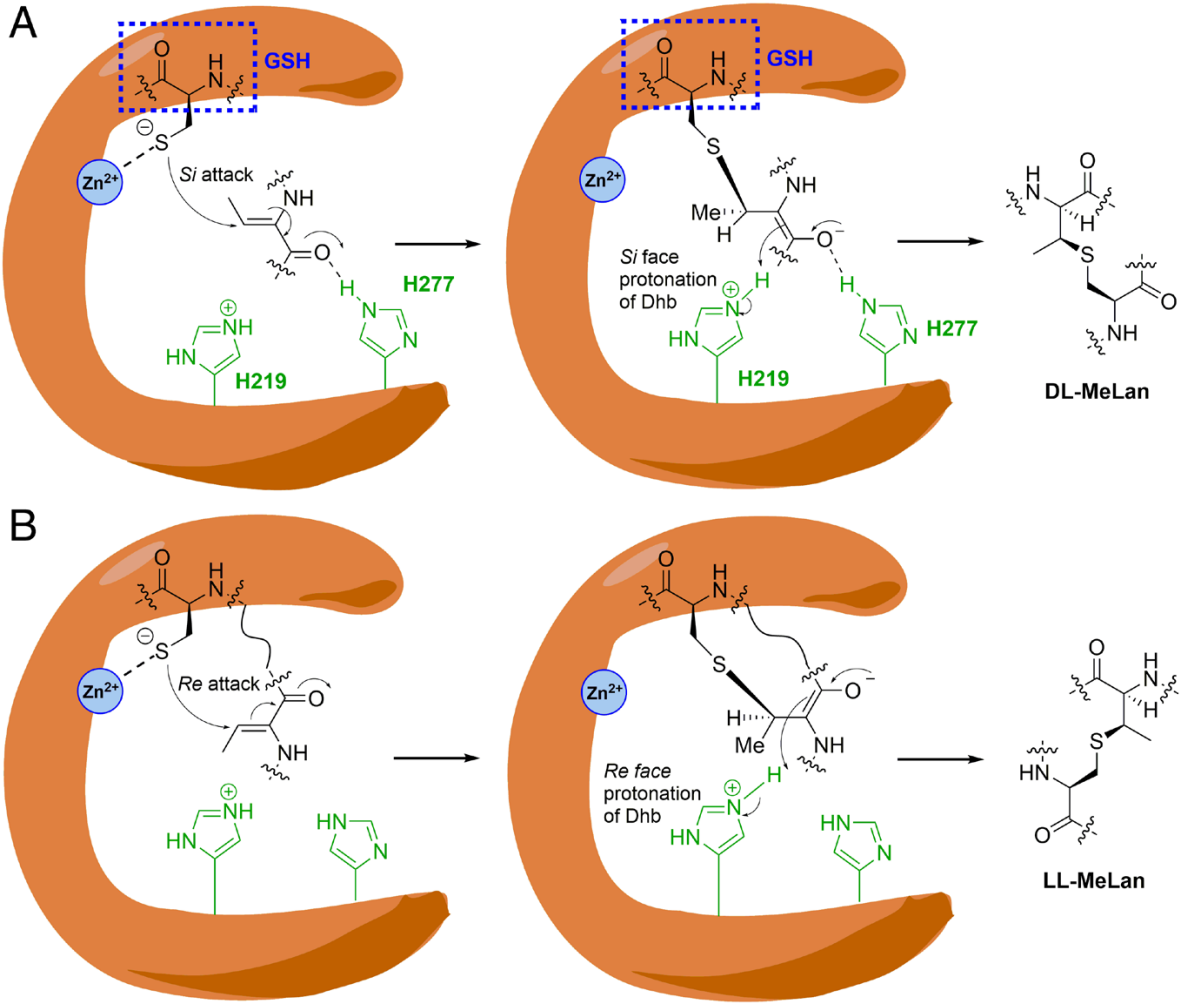
Distinct binding modes of Dhb-containing peptide for the formation of DL-MeLan and LL-MeLan, which are both formed from *anti* addition of the thiol across the double bond but from different faces. *A*. Model for the *anti*-addition of Cys from GSH onto the *Si* face of the β-carbon of Dhb and protonation of the enolate by His219 of LanCL1 on the *Si*-face of the α-carbon of the original Dhb. His277, which could be in the neutral (shown) or protonated imidazolium form, can activate the carbonyl of Dhb and stabilize the enolate intermediate. *B*. During biosynthesis of lanthipeptides, some enzymes catalyze intramolecular addition of Cys onto the *Re* face of Dhb. The overall reaction is also an *anti* addition suggesting the His residue that is equivalent to His219 still protonates the enolate, but residues equivalent to His277 can neither activate the carbonyl of Dhb nor stabilize the enolate oxygen.

The current product structure also provides insight into the manner by which stereochemistry is governed in thia-Michael additions. For the eukaryotic enzymes, the reaction is intermolecular, and the current structure readily explains the experimentally observed *anti* addition (20) that occurs by attack of the thiolate onto on the *Si* face of the β-carbon of *(Z)-*Dhb followed by protonation on the *Si-*face of the α-carbon of the original Dhb by His219 (Fig. 4*A*). For bacterial enzymes involved in lanthipeptide biosynthesis, the conjugate addition is intramolecular, posing additional conformational constraints. Furthermore, several bacterial enzymes including CylM and HalM2 catalyze stereoselective *anti* addition not only to the *Si* face of Dhb for the formation of a subset of macrocycles, but also to the *Re* face for other macrocycles in the same substrate peptide (41–47). This latter outcome is dictated in many lanthipeptides by a specific sequence motif, DhxDhxXxxXxxCys. The LanCL1 structure suggests that the experimentally observed addition of the Cys thiolate to the *Re* face of (*Z*)-Dhb can only be explained by an alternative binding mode of the substrate (Fig. 4*B*). In this alternative binding mode, His277 (or equivalent residues at this position) would no longer be in the correct position to stabilize the enolate oxygen, but His219 could still be the active site acid that protonates the enolate. Based on the known enzymes that catalyze attack on the *Re* face, the identity of the residue at the equivalent position to His277 does not determine selectivity as CylM contains a Lys and HalM2 a His. Previous studies have postulated that the peptide sequence determines which face of the Dhb is presented to the zinc-bound thiolate and not the enzyme. A computational study on the reaction in the absence of enzyme suggested that this preference is dictated by intramolecular hydrogen bonds that selectively lower the activation energy of the reaction for *Re* face attack (42). These computational studies also predicted that such *Re* face attack for the privileged sequences would be faster (have a lower activation energy) than reactions of non-privileged sequences. Thus, a protic residue on the enzyme that can stabilize the enolate may not be needed because of the inherent increased reactivity of the DhxDhxXxxXxxCys motif.

In summary, the current study identified the residue that interacts with the enolate oxygen formed upon thia-Michael addition to dehydroamino acids and explains the selectivity for binding of dehydroamino acid-containing peptides. The data also explain the substrate tolerance of the human LanCL enzymes and may aid in design of inhibitors of bacterial enzymes that make harmful molecules like the enterococcal cytolysin.

## Materials and Methods

### Plasmid Construction.

The LanCL1 gene was previously cloned into pET15b using XhoI and NdeI cloning sites (20). As described in the main text, Cys264 of LanCL1 was mutated to Ala to facilitate crystallographic studies. All the other LanCL1 mutants involved in this study were made by site-directed mutagenesis in the background of this C264A mutation. Using Q5 high-fidelity DNA polymerase (New England Biolabs), the PCR mutagenesis was carried out by an initial denaturing (98 °C for 30 s), then 25 cycles of denaturing (98 °C for 10 s), annealing (65 °C to 72 °C for 30 s), extending (72 °C for 4 min), and a final extending step (72 °C for 5 min). The primers for each mutation are shown in *SI Appendix,* Table S1. The PCR products were purified by QIAquick PCR purification kit (Qiagen) and digested by DpnI (New England Biolabs) at 37 °C for 1 h. The reaction mixture was used to transform *Escherichia coli* DH5α cells using heat shock. The transformed cells were plated on lysogeny broth (LB) with ampicillin agar plates and incubated at 37 °C overnight. Several colonies were picked and inoculated in LB broth with ampicillin (100 µg/mL) at 37 °C overnight. Plasmid extraction was performed by using a QIAprep spin miniprep kit (Qiagen). The identities of the mutant plasmids were confirmed by DNA.

### Expression, Purification, and Crystallization of His_6_-LanCL1.

The pET15b-His_6_-LanCL1 overexpression vector was used to transform *Escherichia coli* Rosetta^TM^(DE3) cells for protein expression. Transformed cells were grown in LB media (supplemented with ampicillin) at 37 °C to OD_600_ = 0.6 to 0.8. Cultures were cooled in an ice bath for 10 min before induction with IPTG at a final concentration of 0.4 mM. Cells were allowed to grow overnight at 18 °C before harvesting. The cell pellet was resuspended in suspension buffer (500 mM NaCl, 20 mM Tris pH 8.0, 10% glycerol).

Cells were lysed by sonication (30-s on-cycles, 20% amplitude, 1 min resting intervals), and the lysates were clarified by centrifugation at 10,000 *g* for 30 min. Clarified lysate was then passed through a 5 mL HisTrap HP column (Cytiva Life Sciences) pre-equilibrated with suspension buffer. The column was washed with eight column volumes (CV) of wash buffer (1 M NaCl, 20 mM Tris pH 8.0, 30 mM imidazole), and His_6_-LanCL1 was eluted from the column on an ÄKTAprime plus system (Cytiva Life Sciences) using a linear gradient from 30 mM imidazole to 250 mM imidazole over eight CV at a flow rate of 2 mL/min. The purest fractions as judged by sodium dodecyl sulfate–polyacrylamide gel electrophoresis were pooled and concentrated down to 5 mL prior to injection onto an 16/60 Superdex® 200 column (Cytiva Life Sciences) equilibrated in 100 mM KCl, 20 mM Tris pH 8.0, 1 mM TCEP. Fractions containing His_6_-LanCL1 were pooled and concentrated to ~20 mg/mL.

Crystallization conditions for His_6_-LanCL1 were determined using sitting-drop sparse matrix screening using the Crystal Gryphon Lipidic Cubic Phase instrument (Art Robbins Instruments). For the initial screen, His_6_-LanCL1 was incubated with 10 mM GSH (Gold Biotechnology). The diffraction quality crystals were obtained by the hanging drop method at 9 °C. Crystals appeared during optimization only when seeding was performed (0.9 μL protein solution, 0.9 μL precipitant solution, 0.2 μL seed stock). Protein samples (~10 to 15 mg/mL) were incubated with 2-5 mM GSH and then mixed with a precipitant solution containing 0.70 to 0.75 M succinic acid pH 7.0, 0.1 M bis-tris-propane pH 7.0. Crystals (thin rods or needles) appeared over the course of 3 to 5 d.

To obtain the GSH-Dhb-Erk bound structure, the GSH cocrystals were soaked into precipitant solution supplemented with 2 to 5 mM of the peptide. Crystals were frozen at time points (30, 60, or 90 min). Crystals were transferred to a cryoprotectant consisting of the reservoir solution supplemented with 20% glycerol prior to vitrification by direct immersion into liquid nitrogen. All crystallographic measurements were collected at Sector 21-ID (LS-CAT, Advanced Photon Source, Argonne National Labs). Data were indexed, scaled, and integrated using autoPROC (48). Crystallographic phases of LanCL1-GSH-Dhb-Erk were obtained by molecular replacement using PHASER as implemented in the CCP4i2 software suite (49) using PDB ID: 3E6U as a search probe. The initial models were refined against the structure factors using REFMAC5 (50), and subsequently manually rebuilt using Coot (51). Models for ligands were only added after the free R factors were below 0.30. For crystallographic and data collection statistics, see *SI Appendix*, Table S3.

## Supplementary Material

Appendix 01 (PDF)

## Data Availability

Structures determined in this study have been uploaded to the PDB: LanCL1 bound to GSH and Dhb-Erk (PDB ID: 8CZK); LanCL1 bound to MeGSH (PDB ID: 8CZL); LanCL1 C264A bound to GSH (PDB ID: 8D0V); native LanCL1 bound to GSH (PDB ID: 8D19).
