# Supplementary material for: The mechanism of thia-Michael addition catalyzed by LanC enzymes
Source: Proc Natl Acad Sci U S A. 2023 Jan 12;120(3):e2217523120. doi: 10.1073/pnas.2217523120 (PMC9934072; doi:10.1073/pnas.2217523120)
Supplement: Supplementary file 1 — Appendix 01 (PDF) [file pnas.2217523120.sapp.pdf]

PNAS

Supporting Information for

The mechanism of thia-Michael addition catalyzed by LanC enzymes.

Chayanid Ongpipattanakul[1,†], Shi Liu[2,†], Youran Luo[2,†], Satish K. Nair[1,2,3,5*] and Wilfred A. van der Donk[1,2,4,5*]

[1]Department of Biochemistry, [2]Department of Chemistry, [3]Center for Biophysics and Computational Biology, [4]Howard Hughes Medical Institute, University of Illinois at Urbana-Champaign, Urbana, IL 61801

[5]Carl R. Woese Institute for Genomic Biology, University of Illinois at Urbana-Champaign, 1206 W. Gregory Drive, Urbana, IL, 61801, USA

[†] Denotes equal contribution

[*] Address correspondence to:

Email: s-nair@life.uiuc.edu ; vddonk@illinois.edu

This PDF file includes:

Supplemental Methods
Figures S1-S12
Tables S1-S5
SI references

Supplemental Methods

Isothermal Titration Calorimetry (ITC) Experiments

Calorimetric analyses were conducted using an Affinity ITC microcalorimeter (TA Instruments) at 25 °C. All buffers used for ITC experiments were degassed prior to use. For binding experiments the cell contained either LanCL1 diluted to 150 μM in a buffer compound of 100 mM KCl, 20 mM Tris pH 8.0, 1 mM TCEP, or NisC or HalM2 diluted to the same concentration in a buffer composed of 1000 mM KCl, 20 mM Tris pH 8.0, 1 mM TCEP. GSH was dissolved to a final concentration of 1.5 mM in the buffer corresponding to the protein being analyzed. The ligand was injected into the cell in 21 consecutive injections (2 μl each) at 300 s intervals. The stirring speed was set to 125 rpm. Data were visualized in Origin 2021b (OriginLab), while nonlinear regression fitting using an independent model was performed using NanoAnalyze (TA Instruments).

Peptide Synthesis

Dha-Erk and (*Z*)-Dhb-Erk

General Peptide Synthesis

Peptides were prepared using a CEM Liberty microwave synthesizer. For automatic synthesis, preloaded Wang resin (0.1 mmol) was deprotected to remove the Fmoc group, and then subjected to repeated cycles of amino acid coupling and Fmoc deprotection. The coupling reactions contained 0.2 M Fmoc-protected amino acid, 1.0 M *N,N′*-diisopropylcarbodiimide (DIC, the activator), and 1.0 M ethyl cyanohydroxyiminoacetate (Oxyma, the activator base) in DMF. The deprotection condition was 20% (v/v) piperidine in DMF. Upon completion of the synthesis, peptides were cleaved from the resin using a cocktail cleavage solution (10 mL for 0.1 mmol resin loading) of 92.5% trifluoroacetic acid (TFA), 2.5 % H_2O, 2.5% dioxa-1,8-octane-dithiol (DODT), and 2.5% triisopropylsilane (TIS). After the cleavage, 70% of the solvent was removed by a gentle nitrogen stream and the remaining solution was added dropwise into 30 mL of ice-cold diethyl ether to obtain a white precipitate that was isolated by centrifugation (2000 xg for 3 min). This procedure was repeated three times. The precipitate was dissolved in H_2O and neutralize to pH 7~8. The solution was filtered and injected onto a RP-HPLC system (Shimadzu) equipped with a Macherey Nagel-C18 HTec column (250 x 10 mm, 5 μm) and eluted using the following conditions: (A: 0.1% TFA in H_2O; B: 0.1% TFA in acetonitrile) 0-30 min, 2-30% B; 30-50 min, 30-50% B. This elution procedure was used as the general HPLC method.

Dha-Erk Synthesis

For Dha-Erk synthesis, cysteine-Erk was synthesized as the precursor peptide. After cleavage from the resin, concentration by solvent evaporation and precipitation with cold diethylether, the white crude product was treated with 10 equiv. of α,α′-di-bromo-adipyl(bis)amide in 20 mL of 100 mM HEPES Buffer/DMF (1:1) as previously reported (1). The suspension was shaken at room temperature for 3 h, and then 37 ℃ for 12 h. Using the general preparative HPLC condition described above, the Dha-Erk eluted at 29-31 min.

(*Z*)-Dhb-Erk Synthesis

For (*Z*)-Dhb-Erk synthesis, pThr-Erk was prepared by solid phase peptide synthesis as the precursor peptide as previous reported (1). After cleavage from the resin and using the isolation procedure described above, the white crude peptide was treated with phosphothreonine lyase

(His_6-SpvC or His_6-OspF) in aqueous buffer (20 mM Tris, 150 mM NaCl, pH 8.0) at 37 ºC until the complete consumption of pThr-Erk as indicated by MALDI-TOF MS (Bruker Ultraflex). Using the general preparative HPLC condition described above, the (*Z*)-Dhb-Erk eluted at 30-32 min.

Acetyl-Dha-Erk Preparation

For acetyl-Dha-Erk synthesis, prior to cleavage from the resin, the cysteine-containing Erk peptide attached to the Wang resin was added to 20 mL of dichloromethane containing 10% acetic anhydride and stirred slowly at room temperature for 2 h to generate acetyl-Cys-Erk. The peptide was cleaved from the resin and isolated as described above. The crude white product was treated with α,α′-di-bromo-adipyl(bis)amide as described above to generate acetyl-Dha-Erk. Using the general preparative HPLC conditions above, the Dha-Erk eluted at 31-33 min.

Fluorescein-Dha-Erk Synthesis

Fluorescein-Dha-Erk was synthesized by treating 1 mg of unprotected Dha-Erk (in 500 μL of 100 mM sodium borate pH 8.4) with 10 equiv. of carboxyfluorescein succinimidyl ester in 500 μL of DMF. The reaction vial was wrapped in aluminum foil, and the reaction mixture was incubated at room temperature for 3 h protected from light, then quenched and 10-fold diluted with 100 mM Tris Buffer pH 8.0. The crude fluorescently labeled peptide solvent was purified by preparative HPLC in the dark. Using the general conditions above, the fluorescein-Dha-Erk eluted at 35-37 min.

Protein Expression and Purification for Kinetic Experiments

WT-LanCL1 and variants were expressed in *E. coli* Rosetta 2 (DE3) cells as N-terminal His_6-tagged proteins. Cells were grown with 100 μg/mL ampicillin in 1 L of autoinduction (AI) media (10 g tryptone, 5 g yeast extract, 5 g NaCl, 3 g KH_2PO_4, 6 g Na_2HPO_4) supplemented with 20 mL of 50X AI sugar solution (25% v/v glycerol, 2.5% w/v glucose, and 10% w/v lactose) at 25 ºC for 20 h. The cell pellet from the above culture was resuspended and lysed in 25 mL of lysis buffer (20 mM Tris, 500 mM NaCl, 1 mM tris(2-carboxyethyl)phosphine (TCEP), 5 mM imidazole, 10% glycerol, pH 8.0). Lysates were centrifuged at 4 °C for 30 min at 75,000 g and supernatants were filtered with centrifugal filters (Fisher Scientific). The resulting lysate was loaded onto a 5-mL HisTrap column (GE Healthcare) packed with Ni-NTA resin for FPLC purification. The column was washed and eluted using solvent A (20 mM Tris-Cl, 500 mM NaCl, 1 mM TCEP, 5 mM imidazole, 10% glycerol, pH 8.0) and solvent B (20 mM Tris-Cl, 500 mM NaCl, 1 mM TCEP, 500 mM imidazole, 10% glycerol, pH 8.0). The loaded column was washed at a flow rate of 1.5 mL/min with a linear gradient from 0 to 20% solvent B over 23 column volumes (CV) and eluted with 100% solvent B for 10 CV. The purity of fractions was determined by SDS-PAGE and Coomassie blue staining. Desired fractions were desalted using a PD-10 desalting column (GE Healthcare) and eluted with storage buffer (20 mM Tris-Cl, 150 mM NaCl, 20% glycerol, pH 8.0). The collected fractions were further concentrated by using a 10 kDa cutoff Amicon ultra centrifugal filter (Millipore) and stored at -80 ºC for further use.

Activity Comparison of LanCL1 Mutants by MALDI-TOF MS Analysis

The qualitative activities of the LanCL1 mutants were compared to that of WT-LanCL1 under the following conditions (***SI Appendix,*** **Fig. S12**): 25 μM Dhb-ERK peptide, 1 mM glutathione, and 2.5 μM LanCL in reaction buffer (25 mM Tris, 150 mM NaCl, 0.1 mM $ZnCl_2$, 1 mM TCEP, pH 8.0). WT LanCL1 or variant were pre-treated with $ZnCl_2$ in Tris buffer for 30 min. TCEP, glutathione,

and Dhb-Erk were subsequently added to the above solution. The reaction mixture was incubated at room temperature up to 24 h. Then 1 µL samples were collected at different time points (1 h, 2 h, and 24 h) and used immediately for MALDI-TOF MS analysis.

Kinetic Study of LanCL1 Mutants by LC/MS-qTOF Analysis

All kinetic studies were performed in triplicate. In 0.2 mL eppendorf tubes was added 5 µL of LanCL quench buffer (1.1 M citric acid and 5.5 mM EDTA) (2). The enzymatic assay contained 1 mM GSH and different concentrations of (*Z*)-Dhb-ERK peptide (25/50/75/100/150/200/250/300 µM) in LanCL reaction buffer (0.5 mM TCEP, 0.1 mM $ZnCl_2$, 150 mM NaCl and 100 mM Tris, pH 7.5). The assay mixture was incubated for 30 min at room temperature. LanCL1 or variants were added to 0.5 µM final concentration. Aliquots were taken from the reaction and added to the tubes with quench solution at defined time points. The quenched samples were analyzed by LC-MS using an EC-C18 2.7 µM column (3.0 × 100 mm Agilent Poroshell 120) attached to an Agilent LC-MS qTOF instrument. The column was eluted with solvent A (100% H_2O, 5 mM ammonium formate) and solvent B (5% H_2O/95% acetonitrile, 5 mM ammonium formate) at a flow rate of 0.40 mL/min. The elution gradient was 30%-60% solvent B over 6 min. The Agilent LC-MS qTOF settings were as follows: ion mode = positive, ion source = Dual AJS ESI, gas temperature = 200 ^oC, drying gas = 13 l/min, nebulizer = 35 psi, sheath gas temperature = 350 ^oC, sheath gas flow = 11 l/min, and MS TOF fragmentor = 125 V. Data were collected over a m/z window of 50-1600 Da in continuous mode with a 0.2 s scan rate using the LC-MS qTOF reference solution kit as internal calibrate standard.

Fluorescent Polarization Binding with Fluorescein-Dha-Erk

All experiments were performed in the same buffer as the kinetic study except that GSH was omitted. Experiments were done in triplicates. Concentration of stock fluorescein-peptides were measured by A_{490} (ε: 70,000 M^{-1} cm^{-1}). To 12 0.2-mL eppendorf tubes was added 50 µL of LanCL reaction buffer and WT LanCL1 was added to a final concentration of 400 µM (300 µM for some of the variants). In a black 96-well plate (Corning 3686), 49 µL of these samples were transferred followed by 1 µL of 1 µM stock fluorescein-labeled Dha-Erk peptide (20 nM final concentration). The plate was covered from light and incubated for 15 min, then fluorescence polarization was measured on a Biotek Synergy H4 hybrid reader. Excitation: emission was 485 nm / 20 nm: 518 nm / 20 nm). Using Origin 2021b, the data were converted to polarization and fitted to the following binding equation (3):

$$y = A1 + (A2 - A1)\frac{(Lt + Kd + x) - \sqrt{(Lt + Kd + x)^2 - 4 * Lt * x}}{2Lt}$$

In which y = measured polarization, *A*1 = minimum polarization, *A*2 = maximum polarization, *Lt* = fluorescent-labeled peptide concentration, and *x* = total enzyme concentration.

Competition Fluorescent Polarization with Acetyl-Dha-Erk

All experiments were done in triplicates. In a black 96-well plate (Corning 3686), 12 of 50 µL mixture (20 µM LanCL1 WT, 20 nM fluorescein-peptide, 1000 µM acetyl-Dha-Erk with 2-fold dilution as final concentration) were prepared and incubated for 15 min before measurement. The data were converted to polarization and fitted to a dose-response function. K_i was calculated using the following equation:

$$K_i = \frac{IC_{50}}{1 + \frac{[L]}{K_d}}$$

Note: [*L*] = fluorescein-peptide concentration

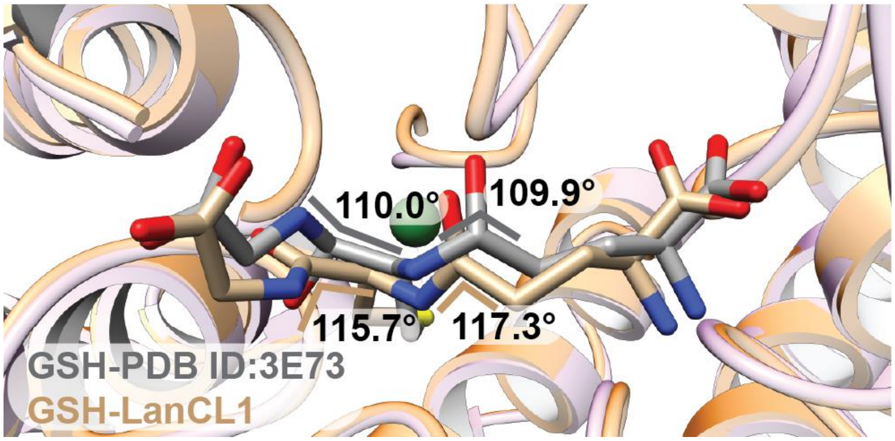

Supplementary Figure S1. Superimposition of the previously reported 2.80 Å structure of GSH-bound LanCL1 (PDB ID: 3E73) with the structure of GSH-bound LanCL1 obtained in this study. Bond angles above the grey arrows correspond to those in the 2.80 Å structure, noting that the amide carbonyl bonds have tetrahedral character. Bond angles below the tan arrows correspond to the structure obtained in this study.

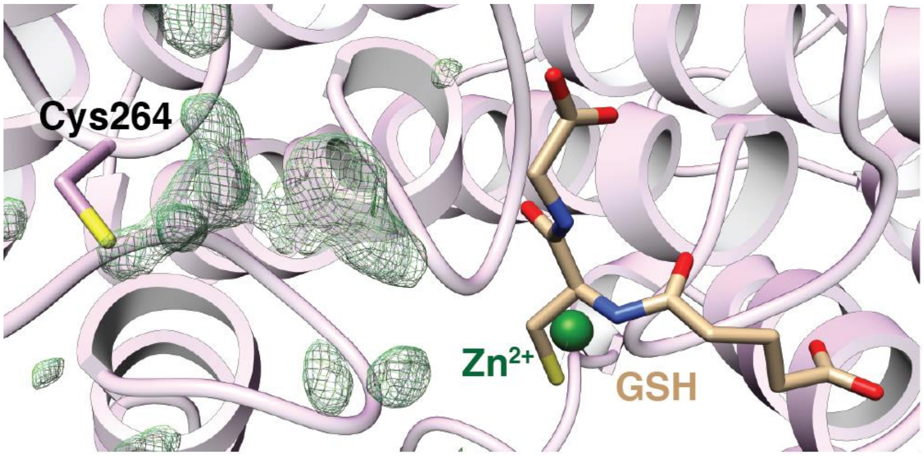

Supplementary Figure S2. Fourier difference map (F_o-F_c), for the region near Cys264 is shown in green (contoured at 3σ above background); note the proximity to bound GSH and Zn^{2+}. As this density was observed during crystallization trials, the C264A variant was constructed and used for biochemical and kinetic analyses.

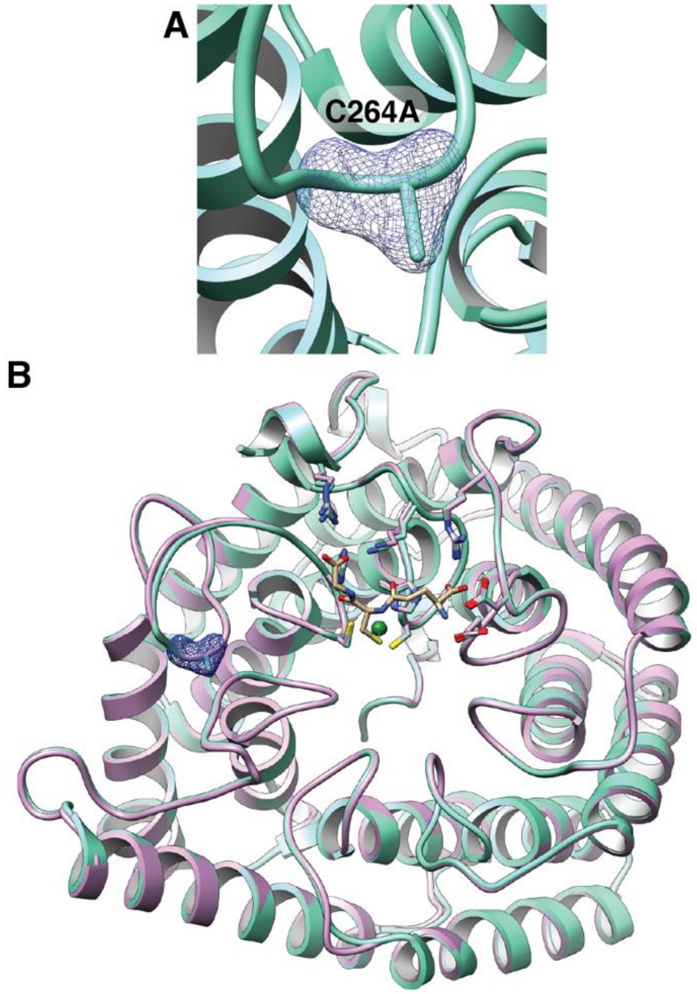

Supplementary Figure S3. (A) Fourier difference map (F_o-F_c) of the LanCL1 variant C264A (blue, contoured at 3σ above background). (B) Superimposition of the LanCL1 C264A GSH bound structure (teal) with the WT LanCL1 GSH bound structure (pink) demonstrating that the mutation does not affect the overall structure of LanCL1, nor the residues that interact with GSH. The ligands from the WT LanCL1 structure are hidden from view.

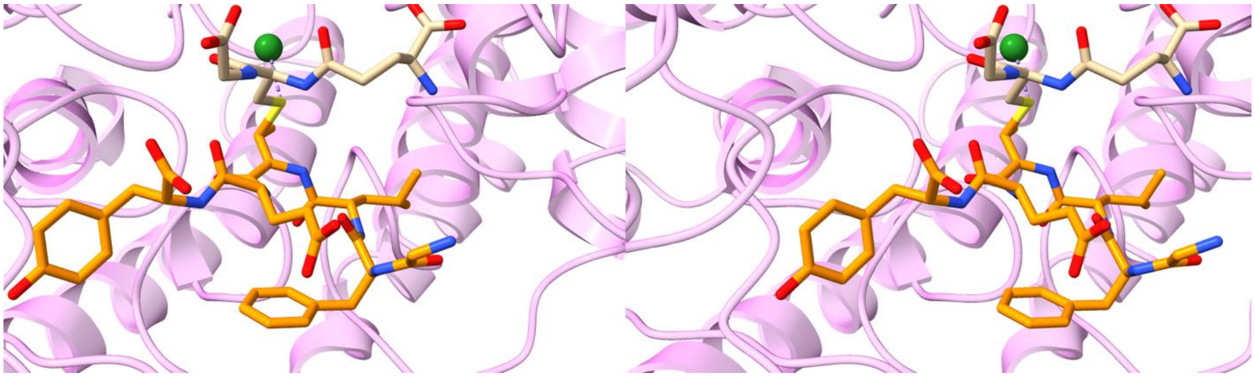

Supplementary Figure S4. Cross-eye stereo view of the GSH-Dhb-Erk product bound to the active site of LanCL1.

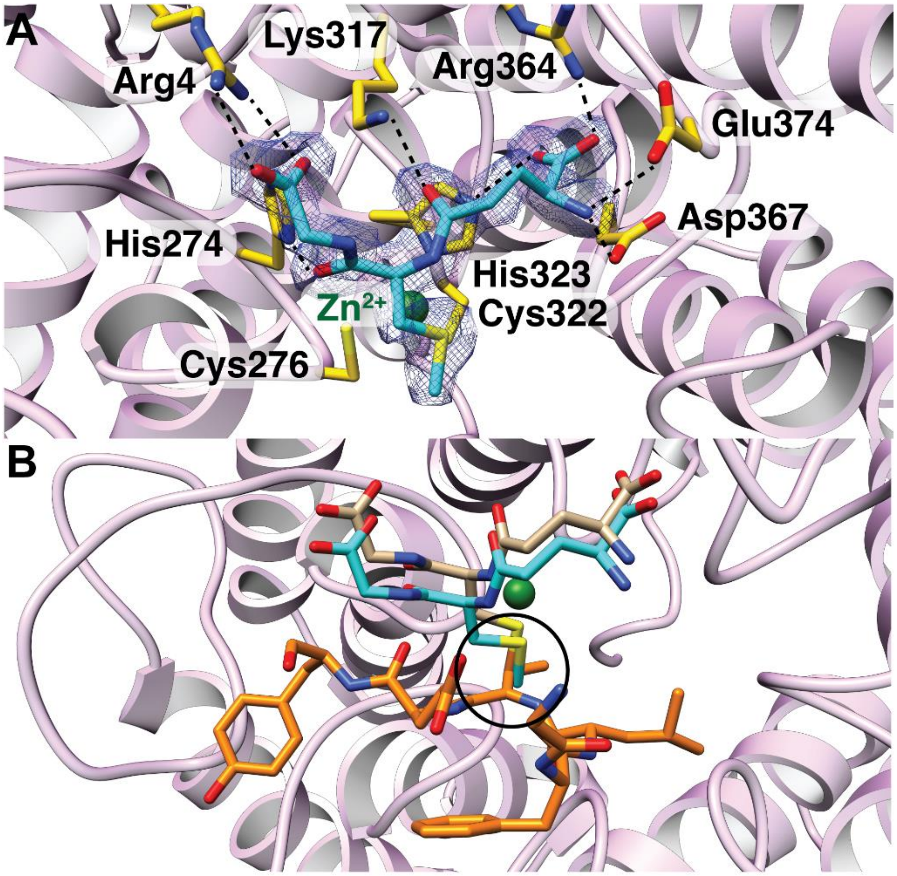

Supplementary Figure S5. (A) Structure of LanCL1 bound to MeGSH showing key hydrogen bonding interactions as dashed lines. LanCL1 residues involved in hydrogen bonding are shown in yellow, while MeGSH is shown in blue. (B) Superimposition of the structure of LanCL1 bound to MeGSH with the structure bound to the GSH-Dhb-Erk product. The position of the methyl group on MeGSH is expected to clash with Dhb-Erk (circle).

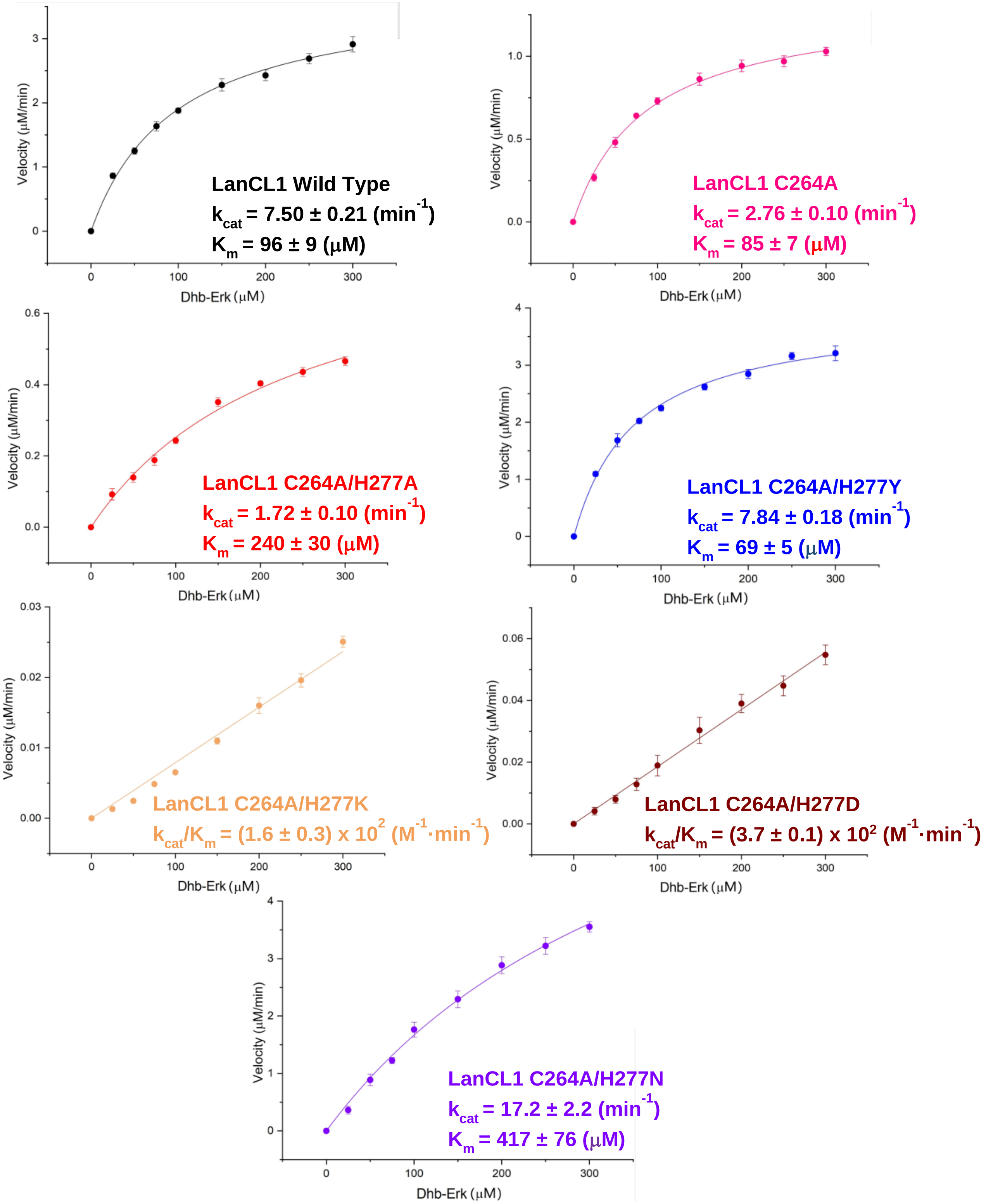

Supplementary Figure S6. Dependence of reaction rate on substrate concentration for LanCL1 and its variants. Initial velocities of glutathionylation of (Z)-Dhb-Erk as measured by LC-MS were fit with the Michaelis-Menten equation or a linear equation (H277K and H277D). Error bars represent standard deviation (n=3 for all data).

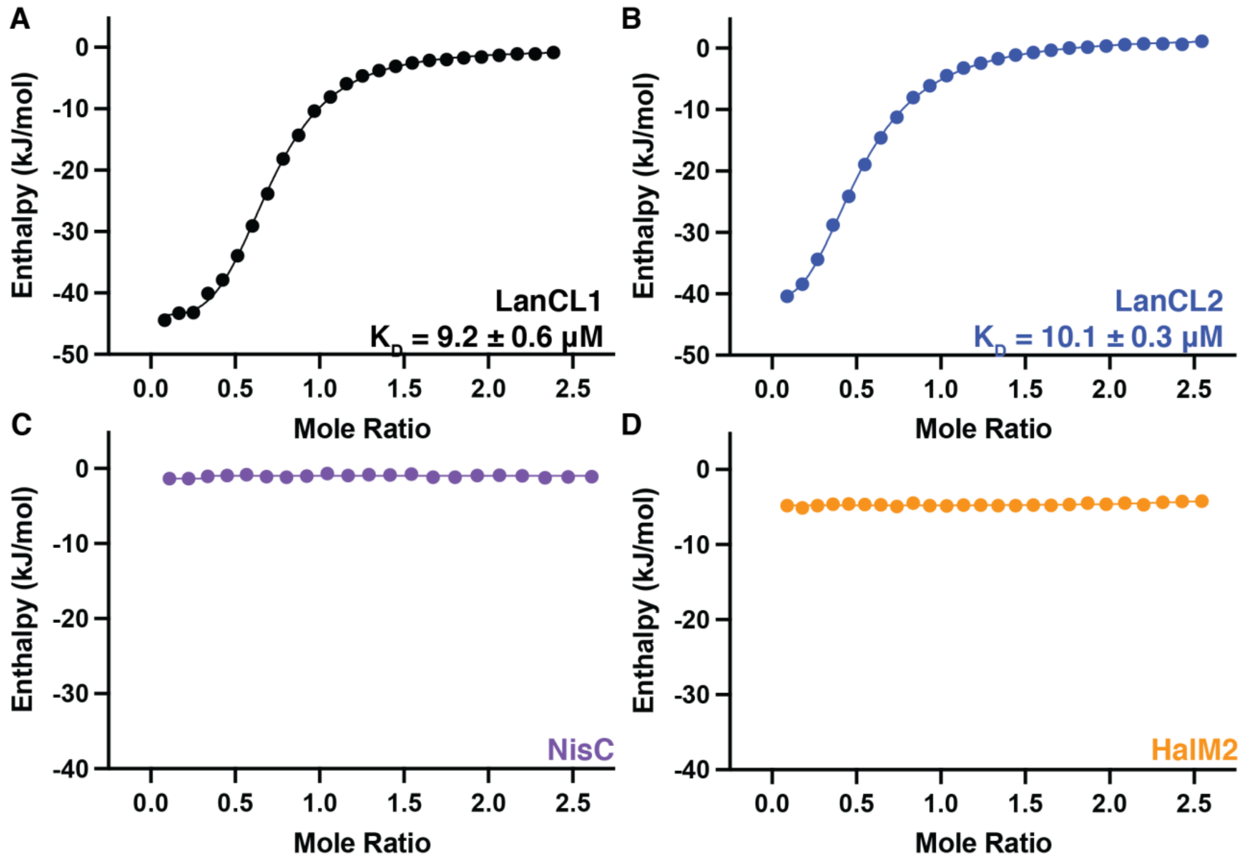

Supplementary Figure S7. ITC binding curves for (A) LanCL1 with GSH, (B) LanCL2 with GSH, (C) NisC and GSH, and (D) HalM2 and GSH.

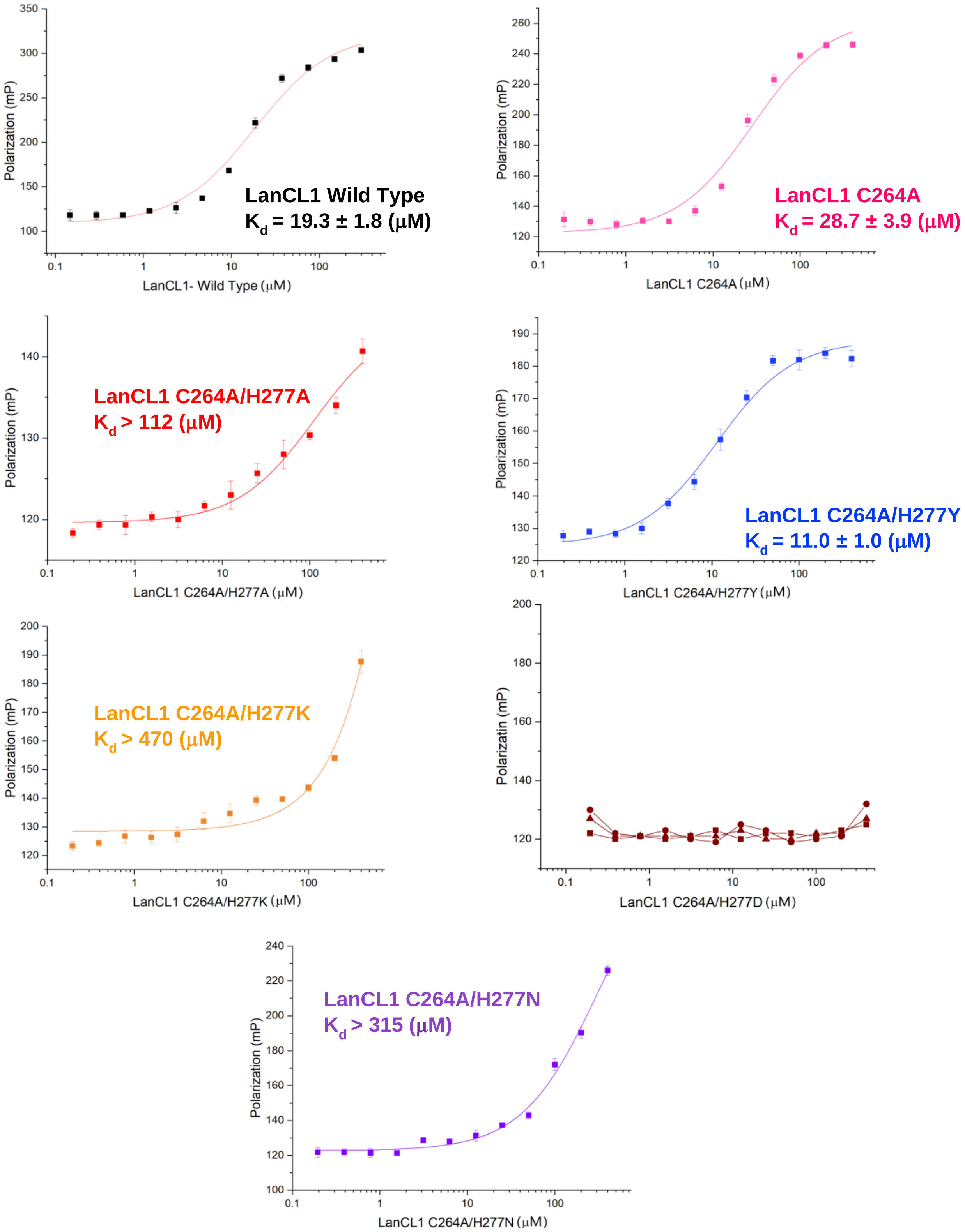

Supplementary Figure S8. Fluorescence polarization plots of fluorescein-Dha-Erk binding with LanCL1 and its variants. Error bars represent standard deviation (n=3 for all data). For some variants where saturation was achieved, the amplitude of the polarization change was smaller than for the wild type protein suggesting different binding modes.

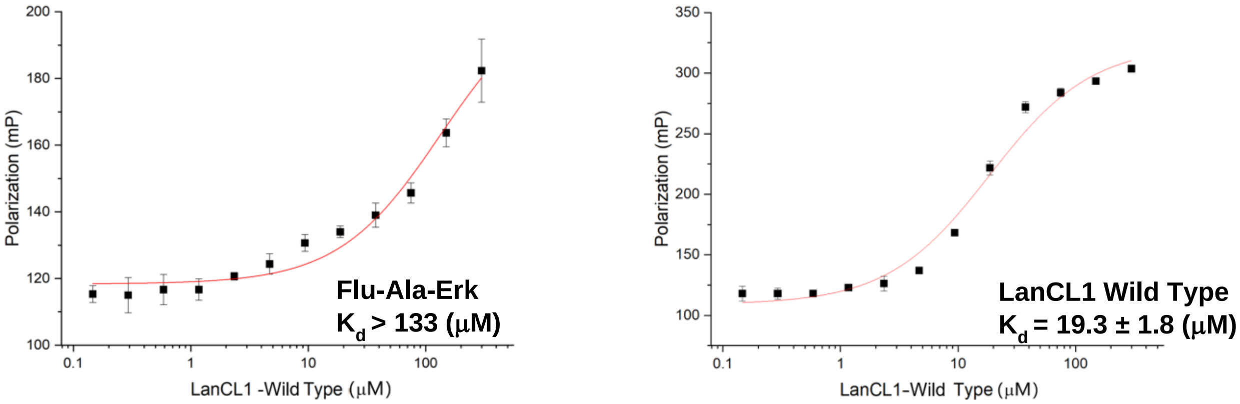

Supplementary Figure S9. Fluorescence polarization plots of binding of fluorescein-Ala-Erk and fluorescein-Dha-Erk to LanCL1. Error bars represent standard deviation (n=3 for all data).

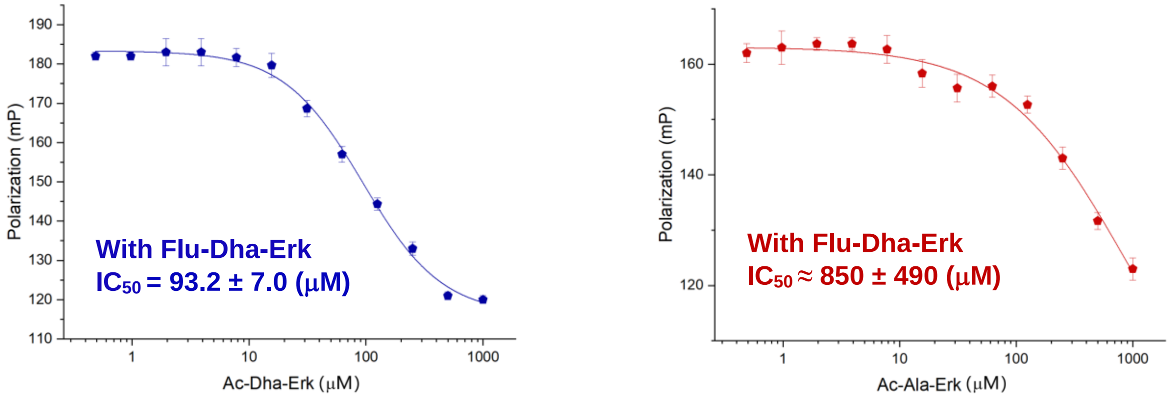

Supplementary Figure S10. Competition fluorescence polarization plots of fluorescein-Dha-Erk binding to LanCL1 and competition by the peptides shown in the plots. Error bars represent standard deviation (n=3 for all data).

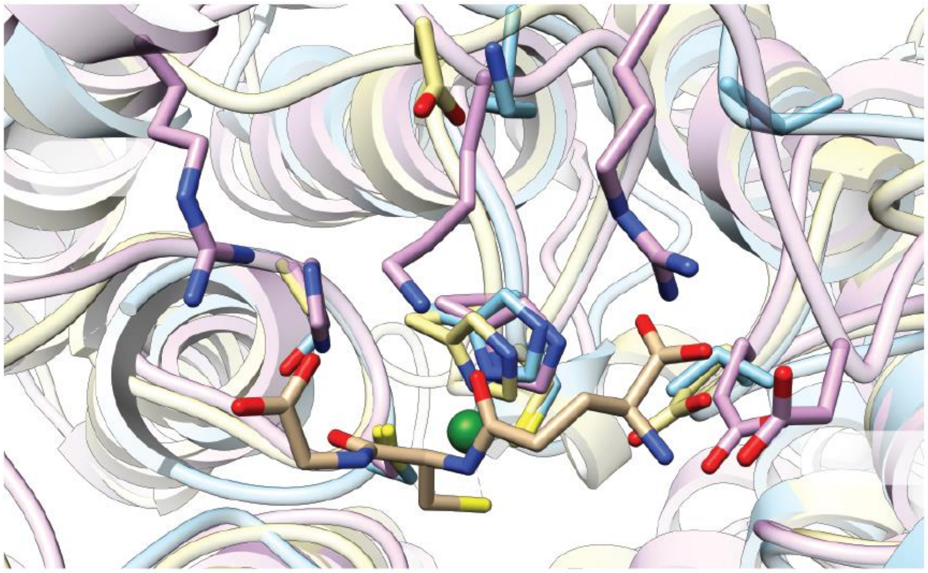

Supplementary Figure S11. Superimposition of the structures of LanCL1 (pink) bound to GSH (tan), NisC (pale yellow), and CylM (blue) demonstrating that NisC and CylM do not share the GSH binding residues that LanCL1 does. The only conserved residues between the three structures are the Zn^{2+} binding residues and the catalytic acid that protonates the enolate.

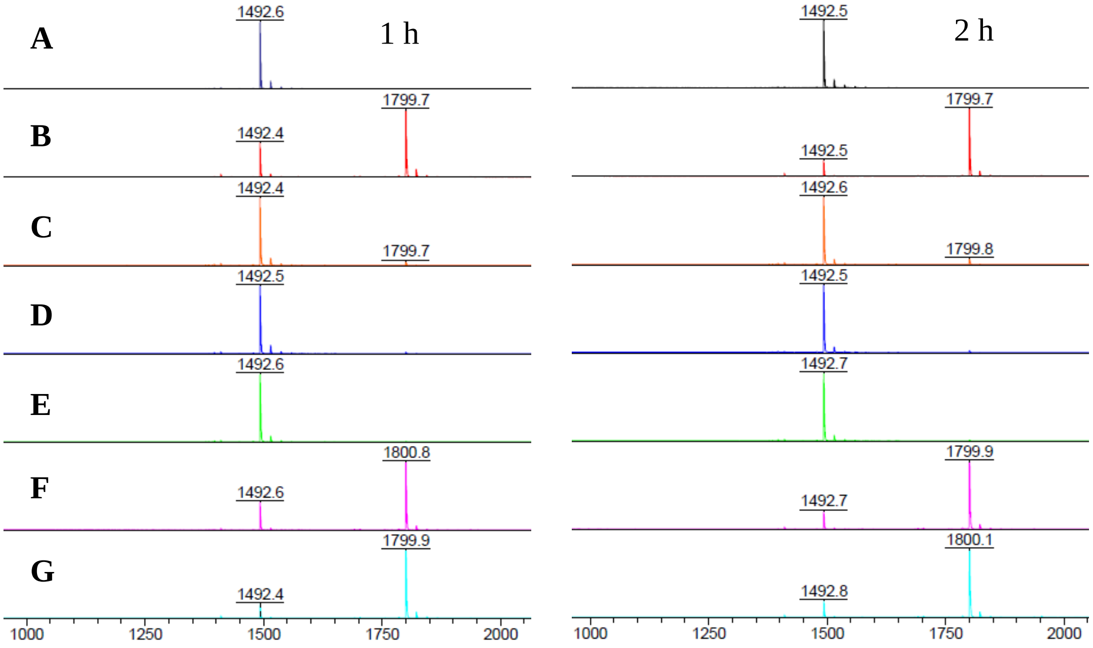

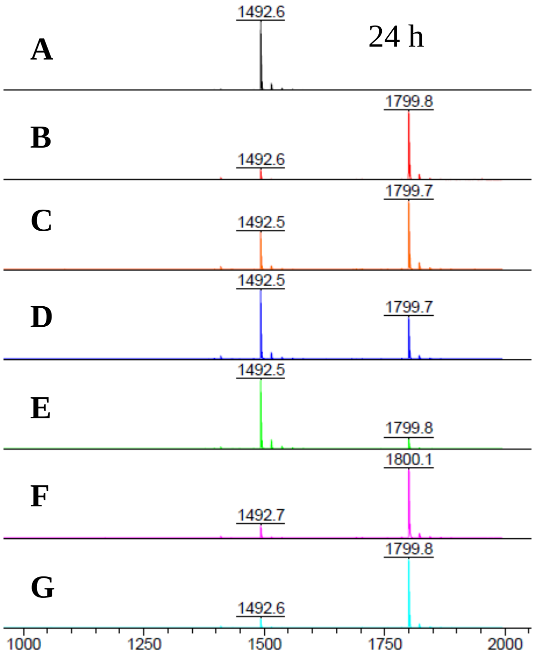

	Enzyme
A	w/o
B	LanCL1
C	LanCL1-C264A/H277A
D	LanCL1-C264A/H277D
E	LanCL1-C264A/H277K
F	LanCL1-C264A/H277N
G	LanCL1-C264A/H277Y

Supplementary Figure S12. Representative MALDI-TOF mass spectra for the reaction of GSH (1 mM) with Dhb-Erk (25 μM) catalyzed by LanCL1 and variants (2.5 μM). The time courses of the reactions with varying concentrations of substrate were then analyzed by quantitative LC-MS.

LanC cyclase homolog (product or substrate)	Motif 1	Motif 2	Accession number
ElxC (epilancin 15X)	LGVS**H**$_{205}$GITG	DAWC**Y**$_{270}$GTPG	FN69435.1
CoiC	NGVA**H**$_{213}$GIGG	PSWC**Y**$_{284}$GTAG	WP_011031311.1
SptC (SapT)	TGMG**H**$_{191}$GVAG	QAWC**Y**$_{263}$GTPG	WP_030776249.1
EpiC (epidermin)	LGLA**H**$_{245}$GILG	NGWC**Y**$_{314}$GDTG	CAA44254.1
MibC (NAI-107)	LGLA**H**$_{253}$GAAG	TAWC**Y**$_{325}$GAPG	ADK32556.1
PedC	FGMA**H**$_{243}$GLPS	VGWC**Y**$_{309}$GDLS	WP_041880928.1
CpinC (pinensin)	LGLA**H**$_{195}$GVPA	LGWC**Y**$_{265}$GDPG	WP_146305898.1
MutC (mutacin II)	VSFA**H**$_{702}$GNSG	ANWC**H**$_{759}$GSTG	AAC38145.1
LctM (lacticin 481)	ASYA**H**$_{725}$GNSG	SQWC**H**$_{782}$GASG	AAC72258.1
ProcM (procholorosins)	LGFS**H**$_{859}$GTAG	ASWC**H**$_{925}$GAPG	WP_011129629.1
VenL (venezuelin)	LGFA**H**$_{751}$GTAG	AYWC**H**$_{811}$GAAG	AEA03262.1
LanM (landornamide)	TGFS**H**$_{897}$GAAG	NTWC**H**$_{961}$GAPG	WP_007357381.1
GarM (actagardine)	GGFS**H**$_{864}$GAAG	ALWC**H**$_{926}$GAAG	ACR33053.1
BovM (bovicin HJ50)	NGFA**H**$_{672}$GISG	GSWC**N**$_{716}$GLLG	ACA51935.2
NukM (nukacin ISK-1)	ASYA**H**$_{721}$GKSG	VSWC**N**$_{778}$GVTG	WP_011152952.1
HalM1 (haloduracin α)	TGFS**H**$_{861}$GVSG	VAWC**H**$_{918}$GAPG	WP_010896633.1
LtnM1 (lacticin 3147 LtnA1)	SGLA**H**$_{793}$GVSQ	TSWC**N**$_{853}$GTSG	AAC56011.1
LtnM2 (lacticin 3147 LtnA2)	FGIA**H**$_{730}$GELG	VGWC**N**$_{775}$GLSG	AAC56013.1
LicM1 (lichenicidin Bliα)	TGFA**H**$_{851}$GTSG	VAWC**H**$_{908}$GAAG	ADW08736.1
LicM2 (lichenicidin Bliβ)	TGFS**H**$_{840}$GLTG	SYWC**H**$_{900}$GAPG	ADW08735.1
GeoM (geobacillins)	AGLA**H**$_{837}$GSSG	SMWC**H**$_{897}$GAAG	WP_011887657.1

Supplementary Table S1. Focused sequence alignment of lanthipeptide cyclase domains showing motif 1 (around LanCL1 His219) and motif 2 (around LanCL2 His77). Names of the associated lanthipeptides are provided in brackets if known; accession numbers of the sequences used in the alignment are also given.

Primer Name	Primer Sequence (5'-3')
LanCL1_H277A_F	gcgccagcgcaccaatggacaagc
LanCL1_H277A_R	ccattggtgcgctggcgcccctg
LanCL1_H277D_F	gcgccatcgcaccaatggacaagc
LanCL1_H277D_R	cattggtgcgatggcgcccctg
LanCL1_H277K_F	gcgcctttgcaccaatggacaagc
LanCL1_H277K_R	cattggtgcaaaggcgcccctg
LanCL1_H277N_F	gcgccattgcaccaatggacaagc
LanCL1_H277N_R	cattggtgcaatggcgcccctg
LanCL1_H277Y_F	gcgccatagcaccaatggacaagc
LanCL1_H277Y_R	cattggtgctatggcgcccctg

Supplementary Table S2. The sequence of primers used to construct LanCL1 mutants.

	LanCL1 GSH•Dhb-Erk	LanCL1 MeGSH	LanCL1-C264 GSH	LanCL1 GSH
**Data collection**				
Space Group	$P2_12_12_1$	$P2_12_12_1$	$P2_12_12_1$	$P2_12_12_1$
Cell: a, b, c (Å)	55.7, 120.6, 144.2	55.5, 120.9, 143.1	55.6, 120.3, 143.0	55.5, 121.1, 142.9
Resolution (Å)[1]	92.5 – 1.91 (1.92 – 1.91)	92.4 – 1.58 (1.61 – 1.58)	92.0 – 1.78 (1.79 – 1.77)	92.4 – 1.51 (1.52 – 1.51)
Total reflections	754,387	1,232,599	861,481	2,175, 475
Unique reflections	75,709	130,838	90,992	150,903
R_{sym} (%)	11.9 (128.4)	8.4 (95.9)	9.9 (108.3)	7.9 (108.8)
R_{pim} (%)[2]	4.1 (43.8)	2.9 (34.8)	3.4 (39.3)	2.2 (34.1)
$CC_{1/2}$[2]	0.998 (0.785)	0.999 (0.800)	0.999 (0.879)	0.999 (0.813)
I/σ(I)	13.5 (2.1)	17.1 (2.1)	13.5 (2.1)	20.6 (2.1)
Completeness (%)	100 (100)	98.3 (95.7)	99.3 (93.7)	100 (100)
Redundancy	10.0 (10.1)	9.4 (8.4)	9.5 (8.2)	14.4 (10.7)
**Refinement**				
Resolution (Å)	25.0 – 1.91	25.0 – 1.58	25.0 – 1.79	25.0 – 1.52
No. reflections	71,871	123,816	85,794	141,284
R_{work} / R_{free}[3]	18.4 / 21.4	18.1 / 20.3	18.1 / 20.1	17.2 / 19.2
**Number of atoms**				
Protein	6,597	6,510	6,487	6,511
Ligands	42	44	42	42
Water	477	656	356	897
**B-factors**				
Protein	31	22	30	20
Water	36	30	36	33
**R.m.s deviations**				
Bond lengths (Å)	0.004	0.006	0.007	0.006
Bond angles (°)	1.08	1.15	1.26	1.11

Supplementary Table S3. Crystallographic and data collection statistics

1. Highest resolution shell is shown in parenthesis.
2. Used as additional indicators of data quality for the highly redundant data sets reported here.
3. R-factor = $\Sigma(|F_{obs}|-k|F_{calc}|)/\Sigma |F_{obs}|$and R-free is the R value for a test set of reflections consisting of a random 5% of the diffraction data not used in refinement.

Supplementary Table S4. Fluorescent polarization experiments with LanCL1 WT and different fluorescein-peptides. Polarization curves are shown in Supplementary Figure S9.

**Fluorescein-peptide**	K_d (μM)
Flu-Dha-Erk	19.3 ± 1.8
Flu-Ala-Erk	79.6 ± 7.6

Supplementary Table S5. Competitional fluorescent polarization of LanCL1 WT with different fluorescein-peptide.

**Fluorescein-peptide**	**Competition peptide**	IC_{50} (μM)	K_i
Flu-Dha-Erk[b]	Ac-Dha-Erk	93.2 ± 7.0	93.1 ± 6.9
Flu-Dha-Erk[b]	Ac-Ala-Erk	850 ± 490	849 ± 489

[b] All fluorescein-peptides were at 20 nM, with LanCL1 WT at 20 μM. The dose-response curves are shown in Supplementary Figure S10.

References

1. Lai KY, *et al.* (2021) LanCLs add glutathione to dehydroamino acids generated at phosphorylated sites in the proteome. *Cell* 184(10):2680-2695.
2. Thibodeaux CJ, Ha T, & van der Donk WA (2014) A price to pay for relaxed substrate specificity: a comparative kinetic analysis of the class II lanthipeptide synthetases ProcM and HalM2. *J. Am. Chem. Soc.* 136(50):17513-17529.
3. Lundblad JR, Laurance M, & Goodman RH (1996) Fluorescence polarization analysis of protein-DNA and protein-protein interactions. *J. Mol. Endocrinol.* 10(6):607-612.
